# Kidney biopsy for renal tubular acidosis: when tissue diagnosis makes a difference

**DOI:** 10.5414/CNCS108412

**Published:** 2015-05-22

**Authors:** Youshay Humayun, Patrick Sanchez, Lindsey T. Norris, Divya Monga, Jack Lewin, Tibor Fülöp

**Affiliations:** 1 Department of Medicine and; 2 Pathology, University of Mississippi Medical Center, Jackson, MS, USA

**Keywords:** allergic interstitial nephritis, distal renal tubular acidosis, hypokalemia, metabolic acidosis, omeprazole, proton-pump inhibitor

## Abstract

Renal tubular acidosis (RTA) is a disorder with variable presentations and oftentimes a nebulous underlying primary diagnosis. We describe a rare cause of RTA as an unusual complication of proton pump inhibitor (PPI) therapy. We report a case of a 33-year-old male with history of hypertension, acid reflux, allergic rhinitis, and low testosterone admitted with complaints of fatigue, weight loss, and unexplained acidosis for ~ 2 months. His medications prior to admission included losartan, omeprazole, potassium chloride, sildenafil, and testosterone propionate injections. His physical exam was unremarkable with a blood pressure of 120/80 mmHg. Initial lab work showed a nonanion gap metabolic acidosis with serum bicarbonate level of 16 mM/L and potassium 3 mM/L. Urine studies showed urine pH of 6.5 and a positive urine anion gap. The serum creatinine was within normal range****(1.13 mg/dL). He required massive doses of bicarbonate and potassium supplementation with minimal improvement of serum chemistries achieved. The cause of apparent distal RTA remained elusive despite extensive blood, urine, and imaging testing. Ultimately a renal biopsy was obtained showing mild to moderate tubule-interstitial inflammation with 5% fibrosis. PPI therapy (omeprazole) was stopped, and he was started on prednisone 60 mg per day. Two weeks later, his RTA findings resolved, and he no longer required bicarbonate and potassium supplementation. Our case highlights the importance of recognizing a unique complication of RTA following PPI therapy. It also underscores the possible need for considering a kidney biopsy in the setting of nondiagnostic laboratory work up to uncover the underlying etiology of RTA and suspected allergic interstitial nephritis (AIN).

## Introduction 

We describe a rare cause of renal tubular acidosis (RTA) as an unusual complication of proton pump inhibitor therapy. The patient presented with primary disturbance of RTA with seemingly normal kidney function. Despite the traditional diagnostic strategies focusing on serum and urine studies, the underlying etiology remained elusive. Ultimately, the patient required a percutaneous kidney biopsy (PKB) to secure the diagnosis of an occult acute interstitial nephritis (AIN) and RTA, likely a consequence of proton pump inhibitor (PPI) exposure. 

## Clinical presentation 

A 33-year-old male with history of hypertension, acid reflux, allergic rhinitis, and low testosterone was admitted with complaints of fatigue, weight loss, and unexplained acidosis for ~ 2 months. He had no exposure to lead, gasoline, paints, adhesives, or glues. His medications prior to admission included losartan, omeprazole, potassium chloride, sildenafil, and testosterone propionate injections. His physical exam was unremarkable, with a blood pressure of 120/80 mmHg. Initial lab work showed a nonanion gap metabolic acidosis with serum bicarbonate level of 16 mM/L and potassium 3 mM/L (Table 1). Urine studies showed urine pH of 6.5 and a positive urine anion gap. Renal function appeared normal with serum creatinine of 1.13 mg/dL, with no previous creatinine levels available at the time of renal consult. No eosinophilia or eosinophiluria was detected. Renal ultrasound remained unrevealing. A 24-hour urine collection showed low calcium excretion (51 mg/24 h) and, as expected, a nondetectable citrate excretion (< 46 mg/24 h). His serologic work up for HIV, Hepatitis B core and surface antibodies, hepatitis-C antibodies, antinuclear antibodies, Anti-Ro, and Anti-La antibodies were also negative. He had no complaints of fatigue, arthralgias, dry eyes/dry mouth, or rashes, suggesting an underlying seronegative rheumatologic disorder. 

**Figure 1.**
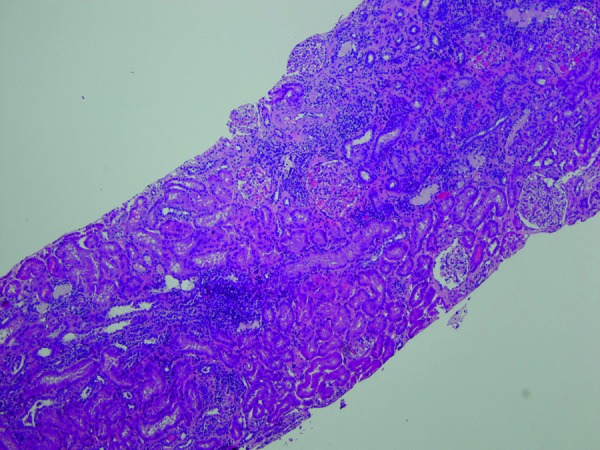
Low power showing interstitial inflammation and area of fibrosis.

**Figure 2.**
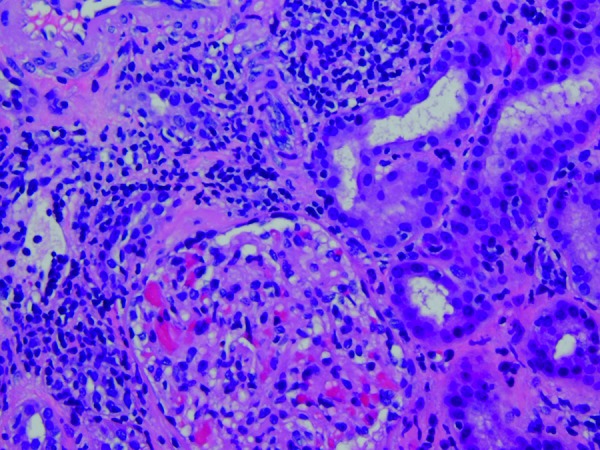
High power of interstitial inflammation and lymphocytic tubulitis in one tubule.

He needed large doses of potassium and sodium citrate supplementation and per os amiloride to restore near-normal serum electrolyte values. Due to diagnostic uncertainty, a renal biopsy was performed, showing findings consistent with AIN and mild to moderate interstitial lymphocytic infiltration with evidence of tubulitis within the cortex. There was also 5% interstitial fibrosis, signifying a chronic component as well (Figure 1 and Figure 2). 

Upon further retrieval and review of past records, his baseline creatinine was 0.6 – 0.8 mg/dL ~ 3 months prior to his hospital presentation. He was also started on omeprazole around that time, suggesting a close temporal relationship between rise in serum creatinine and PPI initiation. In light of the above, his PPI therapy was immediately stopped. Based on retrospective studies [1] and relative safety of short-term steroid therapy, we felt it was reasonable to proceed with a short course of glucocorticoids (oral prednisone 60 mg daily, weaned over 6 weeks). Two weeks, later, during his outpatient follow-up appointment, the RTA was resolved and he no longer required bicarbonate and potassium supplementation. 

## Discussion 

Renal tubular acidosis is a relatively uncommon disorder in adults, which is defined based on different renal pathophysiologic causes of a hyperchloremic (normal anion gap) metabolic acidosis [2]. It is generally classified based on location of injury (proximal tubule or distal tubule), which signifies the primary defect unique to the function of that segment of the nephron. The corollary is that proximal tubule defect (type 2) consists of the inability to reclaim bicarbonate, and the distal tubule has the inability to secrete acid (type 1) or has a hypoaldosterone state (type 4) that drive the nonanion gap acidosis [3]. The etiology of these disorders is very heterogeneous, ranging from infectious, autoimmune, malignancy, metabolic, and structural to drug-induced disorders [2]. Accordingly, clinicians often have to work backwards to determine the primary cause of the problem, and oftentimes the inciting event remains a mystery. This was best exemplified by a retrospective case analysis of 58 distal renal tubular acidosis (dRTA) cases done in Baltimore showing that 55% of them were classified as idiopathic [4]. The work up of dRTA often relies of blood, urine, and noninvasive imaging testing; tissue sampling customarily is not pursued for making the diagnosis. 

While dRTA is a well-described phenomenon of chronic tubulointerstitial disease, it is rarely seen as the sole pathologic finding of AIN. Classically, AIN is often defined as an acute decline in renal function with an inflammatory infiltrate within the kidney interstitium, but it can have more subtle presentations as well. Furthermore, it is known that the “classic” triad of fever, rash, eosinophilia has an insufficient sensitivity and specificity for the diagnosis [5]. Nonetheless, clinicians still use the “classic triad” as the primary means of diagnosis of AIN. As a result, AIN is underreported, and renal biopsies only account for roughly 15% of all acute kidney injuries [5]. In our patient, the cause of dRTA remained obscure despite extensive blood, urine, and imaging testing. It can be concluded that there is insufficient data to describe the full spectrum of renal injury due to either missed diagnosis or lack of histopathologic correlation. Given his normal baseline, we had a high index of suspicion for an anatomic (histological) process to explain this marked and seemingly sudden emergence of biochemical abnormalities. There have been a few case reports describing RTA in settings of acute kidney injury (AKI) secondary to AIN due to medications [6]. There is strong evidence suggesting PPIs, in particular omeprazole, being associated with AIN-related injury. This was best demonstrated by a single-center retrospective study showing biopsy-proven AIN in a setting of PPI therapy [7]. There is little to no data with regards to metabolic acidosis and hypokalemia as presenting cause of PPI-associated AIN. We found one case report suggesting that PPI can worsen acidosis and hypokalemia [8]. The PPIs mechanism of action is inhibiting the H, K ATPase in the stomach for treatment of gastroesophageal reflux disease. Although the kidney also has H, K ATPase channels, it is known to not be affected by the proton pump inhibitor action [9]. PPI are able to inhibit H, K ATPase only at very low PH (< 2), which only occurs in the canaliculi of the parietal cells, but not in the kidney. Therefore, a PPI is extremely unlikely to explain metabolic acidosis based on the “pharmacologic” effect of the medication. There is only a single case report describing a correlation of PPI with worsening metabolic acidosis and hypokalemia [8]. 

The diagnosis of AIN is generally made on clinical suspicion, and, thus, most of the time there is low sensitivity and specificity of making that diagnosis [10]. It is not uncommon for PPI’s to cause an AIN, but it is exceedingly rare for it to present solely as dRTA. As a result, it would be prudent to undertake an intervention that would provide the most benefit with the least potential for adverse effect, such as simply withholding medication. Percutaneous kidney biopsy (PKB) is generally a safe and effective procedure to secure histologic sampling of the renal cortex, included in our institutional experience in the context of graduate medical training [11, 12]. The results of the PKB showed us the definitive finding of AIN as well as some early scarring, making us concerned about potential long-term repercussions of this event (perhaps decades later) [7]. One of the larger retrospective studies done in patients with AIN due to medications showed improved renal recovery with no significant difference in adverse events in patients treated with steroids when compared to the control group [1]. Accordingly, given the potential for benefit and safety of short-term glucocorticoid therapy for AIN [1, 7], we felt it was reasonable to proceed with a short course of prednisone, as described in our report. Our case also underscored the importance of obtaining PKB during work-up of otherwise unexplained RTA [13]. 

## Conclusion 

RTA is condition that can have a variable and subtle presentation. Our case demonstrated that traditional laboratory work-up may not suffice to fully evaluate the underlying etiology. Furthermore, PKB may be warranted to rule out discrete histopathologic injury, such as AIN. Early diagnosis may be crucial in reversing morbidity of an ongoing subclinical injury. 

## Acknowledgment 

At the time of writing this paper, Drs. Youshay Humayun and Lindsey T. Norris are Fellows in Nephrology at the Department of Internal Medicine, University of Mississippi Medical Center. Dr. Patrick Sanchez is a first-year Internal Medicine resident at University of Mississippi Medical Center. 

This material has not been published previously, in whole or part, and is not under consideration for publication elsewhere*. This paper has no tables or figures that would require permission to reprint. The authors have no conflict of interest to declare and accept policies for publication of the paper. 

## Conflict of interest 

The authors have no conflict of interest to declare. 

**Table 1.** Clinical, biochemical, and histological features of the index case.

Variables	On admission	On discharge	2 weeks post discharge
Baseline creatinine, mg/dL	1.13	1.25	0.90
Potassium, mmol/L	3.1	3.7	5.1
CO_2_, mmol/L	14	14	27
Sodium, mmol/L	138	143	134
Chloride, mmol/L	111	111	98
Calcium, mg/dL	8.7	9.3	9.7
